# The costs of infection and resistance as determinants of West Nile virus susceptibility in *Culex *mosquitoes

**DOI:** 10.1186/1472-6785-11-23

**Published:** 2011-10-05

**Authors:** Alexander T Ciota, Linda M Styer, Mark A Meola, Laura D Kramer

**Affiliations:** 1 Wadsworth Center, New York State Dept. of Health. Slingerlands, NY, USA; 2 Department of Biological Sciences, State University of New York, Albany, NY, USA; 3 School of Public Health, State University of New York at Albany, Albany, NY, USA

## Abstract

**Background:**

Understanding the phenotypic consequences of interactions between arthropod-borne viruses (arboviruses) and their mosquito hosts has direct implications for predicting the evolution of these relationships and the potential for changes in epidemiological patterns. Although arboviruses are generally not highly pathogenic to mosquitoes, pathology has at times been noted. Here, in order to evaluate the potential costs of *West Nile virus *(WNV) infection and resistance in a primary WNV vector, and to assess the extent to which virus-vector relationships are species-specific, we performed fitness studies with and without WNV exposure using a highly susceptible *Culex pipiens *mosquito colony. Specifically, we measured and compared survival, fecundity, and feeding rates in bloodfed mosquitoes that were (i) infected following WNV exposure (susceptible), (ii) uninfected following WNV exposure (resistant), or (iii) unexposed.

**Results:**

In contrast to our previous findings with a relatively resistant *Cx. tarsalis *colony, WNV infection did not alter fecundity or blood-feeding behaviour of *Cx. pipiens*, yet results do indicate that resistance to infection is associated with a fitness cost in terms of mosquito survival.

**Conclusions:**

The identification of species-specific differences provides an evolutionary explanation for variability in vector susceptibility to arboviruses and suggests that understanding the costs of infection and resistance are important factors in determining the potential competence of vector populations for arboviruses.

## Background

A comprehensive understanding of the relationships between arthropod fitness and infection with arthropod-borne viral pathogens (arboviruses) is vital to describing the ways in which selective pressures might drive the co-evolution of vector and virus. With the ultimate evolutionary goal of pathogens being dispersal and transmission, and the probabilities of these generally increasing with increased vector fitness, evolution should tend to favor an innocuous relationship between arboviruses and their mosquito hosts [1]. Indeed, many studies have demonstrated that such a benign association often exists [2-4]. This is particularly the case with viruses which rely more extensively on vertical transmission for maintenance [5]. Despite this, evolution away from virulence is not always the rule if, as initially proposed in the trade-off hypothesis, increased virulence is itself coupled with increased transmission [6,7]. Specifically, increases in transmissibility are generally associated with increased replication, yet increased replication may be inherently coupled with increased virulence. Additional strategies such as infection causing increases in feeding rate as documented for malaria [8] and *West Nile virus *(WNV;[9]) may partially overcome the costs of virulence on pathogen transmission, yet ultimately pathogen evolution should move towards the balance between virulence and pathogen load which maximizes transmission.

On the host side, evolution should independently favor resistance and/or tolerance to pathogenic viruses, yet these immune defences are often associated with their own fitness costs, so predicting the way in which host-pathogen interactions will affect the evolution of host response requires a detailed understanding of the balance between the costs of infection and immunity in individual species.

*West Nile virus *is the most widespread arbovirus in the United States and is primarily vectored by *Culex *mosquitoes including *Cx. pipiens *in the northeast and north central, *Cx. tarsalis *in the west, and *Cx. quinquefasciatus *in the south and southwest USA [10,11]. Although all species have the capacity to support high levels of replication of WNV, vector competence differs among and within species [12-14]. Histopathology of WNV in colonized *Cx. quinquefasciatus *demonstrated that infection can be associated with significant tissue damage in salivary glands [15] and apoptosis of midgut cells[16]. Similar pathology has been noted in mosquito vectors during infection with other arboviruses including *Semliki Forest virus *[17] and *Western equine encephalomyelitis virus *[18]. Additionally, studies monitoring life history traits have also demonstrated that fitness costs in terms of both survival and fecundity are often associated with arbovirus infection in mosquitoes, particularly in the case of alphaviruses [19-22]. A previous study in our laboratory found that decreased fecundity but not survival was associated with infection of WNV in *Cx. tarsalis *mosquitoes [9]. Here, in order to evaluate the extent to which virus-vector relationships are species-specific, we performed similar studies in *Cx. pipiens *mosquitoes. Specifically, we measured and compared survival, fecundity and feeding rates in bloodfed mosquitoes that were (i) infected following WNV exposure (susceptible), (ii) uninfected following WNV exposure (resistant), or (iii) unexposed. We hypothesized that differences in the costs of both infection and resistance among mosquito species may partially explain differences in susceptibility in nature.

## Results

### WNV infection rates and viral titers

**Figure 1:**
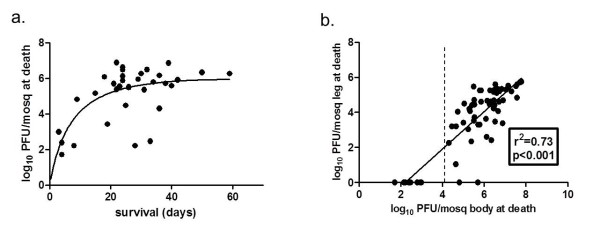
**WNV titers in *Cx. pipiens *mosquitoes at the time of death**. (a) Data points represent individual mosquitoes and the line represents the best-fit relationship between WNV body titer and time of death. (b) Relationship between WNV body titer and WNV leg titer in individual mosquitoes. The dotted line refers to the threshold body titer required for WNV dissemination (4.2 log_10 _pfu) and the linear regression analysis was completed for all values above that threshold.

The titer of WNV in the serum of infected chicks fed upon by mosquitoes was 10^6.3 ^and 10^6.7 ^log_10 _pfu/ml for replicates I and II, respectively. Infection rates were high for exposed groups in both replicates, with 36 of 42 fully-engorged mosquitoes infected in replicate I and 32 of 39 infected in replicate II. This translated to a combined infection rate of 84.0% and therefore relatively small numbers of resistant mosquitoes. As expected, WNV titers in mosquito bodies increased with time following infectious bloodmeal until approximately day 20, yet there was also substantial variation among titers which is not explained by time (Figure 1a). The proportions of infected mosquitoes with disseminated infection (i.e. WNV + legs) were 83.3% and 87.5% for replicates I and II, respectively. If mosquitoes that died prior to day 5 are omitted (due to low probability of dissemination), the combined proportion of disseminated infections among infected mosquitoes increases to 91.2%. WNV titer in legs was highly positively correlated with WNV body titer (r^2 ^= 0.73, p < 0.001), yet the threshold viral load required for WNV dissemination was 4.2 log_10 _pfu/mosquito body (Figure 1b).

### Survival and Wing length

**Figure 2:**
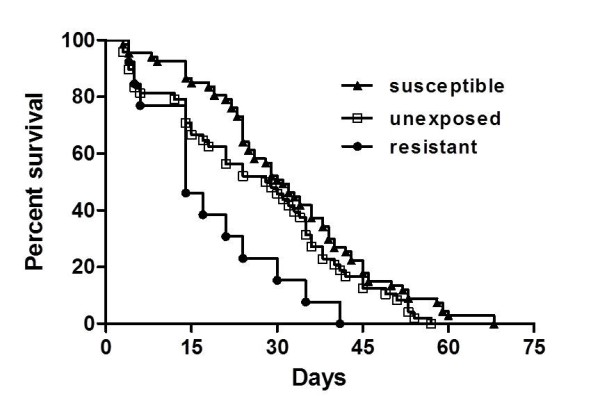
**Combined survival of individual groups of *Cx. pipiens *following initial bloodfeeding**. Statistically significant differences in survival were measured between both WNV susceptible and unexposed groups relative to WNV resistant mosquitoes (log-rank, p < 0.001).

*Cx. pipiens *survival curves were generated and compared for susceptible, resistant and unexposed groups using both individual replicate and combined data. Survival was significantly decreased in resistant groups relative to both susceptible and unexposed groups in both replicates (p < 0.01, log-rank; Figure 2). Survival time was on average 9.7 days (replicate I) or 14.5 days (replicate II) less in resistant relative to susceptible mosquitoes. Although in both replicates, average, median, and maximum survival times were also higher in susceptible groups relative to unexposed groups (table 1), a difference in survival between these two groups was only significant for replicate I (p < 0.01, log-rank). In addition, significantly increased survival occurred for both susceptible and unexposed groups between replicates (table 1). The reason for the difference between replicates in not clear, although variations in colony fitness between generations are not uncommon.

**Table 1:** Summary statistics for *Cx. pipiens *fitness following feeding on a bloodmeal with (resistant, susceptible) or without (unexposed) WNV

	replicate 1			replicate 2		
	**Resist**.	**Suscept**.	**Unexp**.	**Resist**.	**Suscept**.	**Unexp**.
N	6	36	22	7	32	14
Wing (mm)	3.67	3.73	3.69	3.70	3.72	3.67
AST^1^(d)	16.8	26.5	20.9	19.7	38.2	34.2
MST^2^(d)	13.5	25.5	21.0	14.0	39.5	36.0
MaxST^3^(d)	35.0	59.0	45.0	41.0	68.0	57.0
Rafts/fem^4^	0.46	1.39	1.32	0.86	1.25	1.29
Eggs/raft	174.0	162.1	151.7	152.2	144.5	140.1
R_0_^5^	37.1	94.6	79.1	65.2	87.1	88.2
T^6^	14.2	15.0	16.8	13.4	21.2	23.6
r^7^	0.25	0.30	0.26	0.31	0.21	0.19

^1 ^average survival time

^2 ^median survival time

^3 ^maximum survival

^4 ^mean total egg rafts produced per female

^5 ^net reproduction rate

^6 ^mean generation time

^7 ^population growth rate

Mean wing lengths between replicates and among groups were statistically similar for all individual comparisons (p > 0.05, t-test). Values for wing length ranged from 3.21 mm to 4.00 mm with a combined mean length of 3.70 mm (table 1).

### Fecundity

**Figure 3:**
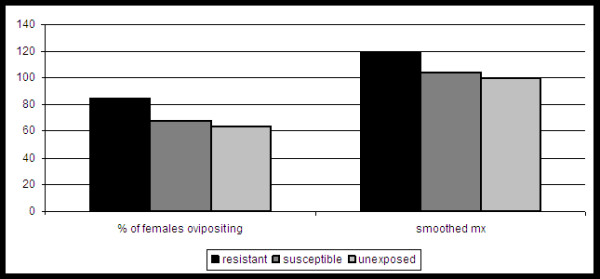
**Combined fecundity of individual groups of *Cx. pipiens***. The percent of females ovipositing refers to individuals producing at least one egg raft during the study and smoothed m_x _refers to the average daily reproductive output while surviving. No significant differences were measured in these statistics (fisher's exact, p > 0.05).

**Figure 4:**
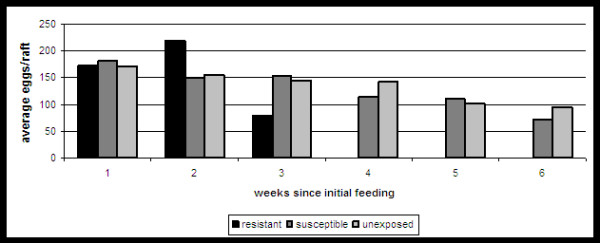
**Mean egg raft sizes produced by individual groups of *Cx. pipiens *during the study**. No mosquitoes in the resistant group survived beyond week 3 of the study.

**Figure 5:**
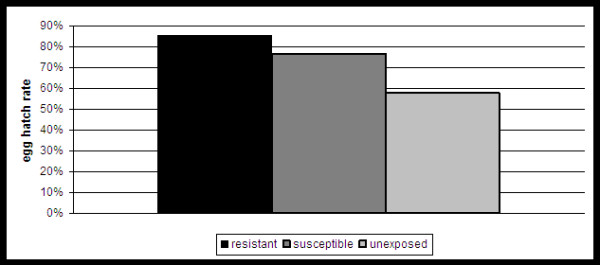
**Combined percent of *Cx. pipiens *egg rafts hatched throughout the study**. Significant differences were measured between groups (chi-squared, p < 0.001).

The reproductive output of each group was monitored and compared throughout the study. Although the values for R_0 _are substantially lower for the resistant group in both replicates, this can be wholly attributed to the decreased survival observed with this group (table 1). In fact, values for both r and T demonstrate that the rate of increase of the resistant population is similar or slightly greater than both other groups while surviving (table 1). In addition, although lacking in statistical significance due to low sample size, both smoothed m_x _and total % of females laying eggs were modestly larger for the resistant group when replicates are combined (Figure 3; chi-squared; p > 0.01). This difference cannot be attributed to WNV infection as all measures of egg output were similar among susceptible and unexposed groups (table 1; Figure 3). A gradual decline in reproductive output as noted by the number of eggs/raft was measured throughout the study (Figure 4). An exception to this was seen with a spike in eggs in the resistant group at week 2, yet this data point represents just a single raft containing 218 eggs. Significant differences were measured in the egg hatch rates among groups (Figure 5; chi-squared, p < 0.001). Again, there is no apparent indication that WNV infection is associated with a decreased egg hatch rate, as rates for the unexposed group were in fact significantly lower than both susceptible and resistant groups.

### Bloodfeeding behaviour

The combined mean weekly feeding rate for all experimental mosquitoes was 31.65% and was statistically similar among groups (table 2; fisher's exact, p > 0.05). The percent of female *Cx. pipiens *not taking a subsequent bloodmeal following the initial feeding to enter the study was 28.4% (table 2). Although this again did not differ statistically among groups, it is notable that the lowest percentage of unfed females was observed in the resistant groups despite significantly decreased survival (table 2; fisher's exact, p > 0.05). Although a higher proportion of females fed, levels of engorgement were also on average lower in the resistant groups based on mean qualitative scores (table 2). Statistical significance was also not attained here when combined resistant groups were compared to both combined susceptible and unexposed groups (t-test, p > 0.05) yet this likely was due to the combination of small sample size and large deviations in the resistant group. In addition, since the statistical power was based solely on averaging qualitative observations, results do not rule out the potential for this difference to be biologically significant.

**Table 2:** Summary of blood feeding behavior among study groups

	**weekly feeding rate**^**1**^	**total unfed**^**2**^	**engorgement score**^**3**^
resistant	34.21%	23.1%	2.0 +/- 1.3
susceptible	29.93%	28.8%	2.6 +/- 1.3
unexposed	34.15%	29.7%	2.5 +/- 1.1

Data represents combined values from two replicates.

^1 ^Mean weekly feeding rates for surviving mosquitoes

^2 ^Initial bloodmeal feeding was required for entry into the study. Percents represent individuals that did not take at least one subsequent bloodmeal.

^3 ^Mean score +/- SD. Levels of engorgement were qualitatively scored as 0-4, with 0 representing no blood and 4 representing full engorgement.

## Discussion

Unlike the acute, lytic, and often pathogenic infections which are associated with arbovirus infection of vertebrates, infections of mosquito vectors are often persistent and generally thought to be largely benign [1]. While it is true that the primary vectors of arboviruses have the capacity to support high levels of virus replication, it is not always the case that mosquito hosts are free from fitness costs as a result of these infections [5]. In addition, recent studies demonstrate that mosquito immune responses to arbovirus infections are measurable and complex, suggesting that an evolutionary need to retain such a defence system likely exists [23]. Although the costs of immune defence have been documented with other insect-pathogen relationships [24-27], such costs generally remain uncharacterized for arbovirus-mosquito associations. Defining the costs of both defence and infection has direct implications for predicting the evolution of virus-vector interactions and, consequently, for forecasting the success of emerging vector-borne diseases.

*West Nile virus *emergence and subsequent expansion in the U.S. has been driven by the availability of both highly susceptible avian hosts and highly competent mosquito vectors in the *Culex *genus [11,28]. While WNV strain has a significant impact on phenotype, studies have also clearly demonstrated that, although *Cx. quinquefasciatus*, *Cx. pipiens*, and *Cx. tarsalis *all have the capacity to maintain WNV activity, vector competence differs among and within each species [13,14]. Consistent with these findings, colonies of *Cx. tarsalis *and *Cx. pipiens *in our laboratory differ substantially in competence, with *Cx. pipiens *being more susceptible to WNV infection and *Cx. tarsalis *being more likely to transmit once infected [29]. Both susceptibility and tolerance to infection will directly affect vector competence and are inherently linked to the cost of arbovirus infection and/or immune defence in the mosquito. For this reason, it is reasonable to assume that differences in vector competence may be partially explained by the costs of infection or defence. A previous study in our laboratory with *Cx. tarsalis *demonstrated that infection with WNV resulted in decreased fecundity of mosquitoes [9]. In addition, this study demonstrated that a fitness cost was not associated with resistance to infection. Taken together, these results could partially explain the maintenance of a relatively high number of WNV resistant *Cx. tarsalis *in this population. Here, we evaluated survival, fecundity and feeding rates in *Cx. pipiens *in groups which were (i) susceptible, (ii) resistant, or (iii) unexposed. Based on the assumption that the costs of resistance and infection can partially explain patterns of vector competence in nature, we hypothesized that the increased susceptibility of *Cx. pipiens *to WNV should be reflected in these costs.

Results clearly demonstrate that costs are indeed species-specific. Unlike *Cx. tarsalis*, there was no evidence of decreased fecundity with WNV infection of *Cx. pipiens *as measured by eggs/female (table 1), eggs/raft (table 1; Figure 4), or percent of females ovipositing (Figure 4). In addition, hatch rate was in fact significantly higher in the infected group relative to the unexposed group (fisher's exact, p < 0.05; Figure 5). Although egg ovipositing females are likely to be mated, whether differences in hatch rate can truly be attributed to WNV infection cannot be fully assessed unless the reproductive status of individual females was known. In addition, hatch rates for resistant mosquitoes was significantly higher than both susceptible and unexposed groups (fisher's exact, p < 0.05; Figure 5). This difference can likely be attributed to the fact that eggs in primary rafts were more likely to be viable than those in subsequent rafts and that resistant individuals rarely survived long enough to produce more than a single egg raft (table 1, Figures 1 and 4). Overall, survival time was significantly decreased for resistant individuals as compared to both susceptible and unexposed groups (log-rank, p < 0.01; Table 1; Figure 1). Combined median survival time for resistant groups was just 14 days, compared to 25.5 or 39.5 days for susceptible mosquitoes in replicates I and II, respectively (table 1, Figure 1). Although generational fitness differences in mosquito colonies are apparent here, both combined and individual survival data demonstrate significantly decreased survival for resistant mosquitoes relative to the more abundant susceptible population. In addition, although significant differences in survival were not identified between susceptible and unexposed groups, average, median and maximum survival time was lower in unexposed groups for both combined and individual replicate data (table 1; Figure 1). Since the unexposed group would be expected to be composed of a majority of susceptible mosquitoes together with a small percentage of less fit, resistant mosquitoes, this result is also consistent with the overall conclusion that WNV resistance is associated with decreased survival. Since the majority of mosquitoes were susceptible, sample sizes for resistant groups were small; but the fact that this difference in survival was observed in both replicates increases confidence in this finding. Differences in survival both among replicates and groups cannot be attributed to size, as wing sizes for all mosquito groups tested were statistically equivalent (table 1). The combined infection rate of 84.0% is comparable to previous studies with WNV and, as expected, was significantly higher than that measured with *Cx. tarsalis *using the same virus strain and experimental conditions (33.3%; fisher's exact, p < 0.0001; [9]). This confirms that these mosquitoes differ substantially in their capacity to maintain resistant individuals in the population and that these differences are consistent with the cost of resistance and infection now identified between these two species. Specifically, *Cx. pipiens *display no cost from infection yet a significant cost for resistance, which predicts that more susceptible individuals will be maintained in the population, while *Cx. tarsalis *display no cost for resistance yet a significant cost for infection, which predicts more resistant individuals will be maintained in the population. The bloodfeeding results are also consistent with these differences, as the increased feeding rate identified with WNV infection of *Cx. tarsalis *by Styer et al can be viewed either as compensatory effect selected by the virus to overcome the cost of infection in this host or, more likely, a host reaction to decreased fecundity associated with WNV infection. In either case, a species that does not accrue such costs from WNV infection should, in turn, not develop increased feeding rates with infection. Indeed, results here demonstrate that no differences in feeding rates exist among experimental groups of *Cx. pipiens *(table 2).

What remains unclear is how well these colonized mosquitoes represent the variation within populations of *Culex *mosquitoes in nature. Clearly, genetic heterogeneity will be much greater in nature, and this will have significant implications for phenotypic variation in terms of susceptibility. In addition, the premise that population structure has been significantly impacted by infection status assumes that both prevalence of infection and time of co-evolution with WNV have been sufficient for such relationships to evolve. Although the co-evolutionary history of WNV and *Culex *mosquito populations in the U.S. spans back just over a decade, *Cx. pipiens*, unlike *Cx. tarsalis*, are an invasive species which likely have a more historic association with WNV [30]. This could potentially explain the increased tolerance to infection in *Cx. pipiens *identified here, yet these relationships are likely much more complex, as many populations of *Cx. tarsalis *are highly competent vectors and small variations in the genetic signature among *Cx. pipiens *can significantly alter susceptibility [14]. Alternatively, these relationships could be generic responses to a host of pathogens as *Culex *mosquitoes in the U.S. have had more historic relationships with other arboviruses including *St. Louis encephalitis virus*, a flavivirus which is closely related to WNV [31]. Future studies will help elucidate the specificity of the costs of infection and resistance.

In addition, it would be naïve to assume that all differences in arbovirus susceptibility and competence of individual mosquito species or populations can be explained exclusively by the costs of resistance and infection, as the capacity of an arbovirus to infect, replicate and disseminate in particular hosts will likely be largely due to virus-dependent molecular interactions beyond host immunity, such as the efficiency of recognition and entry, and replication in particular hosts. In addition, the relationship between temperature and vector competence is well established [32]. Despite this, results here clearly demonstrate that the costs of infection and resistance in vector populations are important factors to be considered when both predicting the way in which current vector-virus relationships might co-evolve and assessing the potential for arboviral emergence in previously naïve vector populations.

## Conclusions

In contrast to our previous findings with a relatively resistant *Cx. tarsalis *colony, WNV infection did not alter fecundity or blood-feeding behaviour of *Cx. pipiens*, indicating that there is no cost associated with WNV infection in this population. Further studies will help clarify if additional WNV adaptation to a vector population could lead to increased virulence in these vectors and therefore constrain the evolution of more efficient strains. In addition, results clearly demonstrate a significant decrease in survival associated with WNV resistance under our experimental conditions. The identification of species-specific differences provides an evolutionary explanation for variability in susceptibility of mosquito vectors to arboviruses and suggests that understanding the costs of infection and resistance, together with the co-evolutionary history of vector and virus, are important considerations when evaluating the potential competence of vector populations for arboviruses.

## Methods

### Virus strains and testing

WNV used for experimentation was derived from WNV NY003356, isolated from an American crow in 2000 from Staten Island, NY [33] and prepared by three rounds of plaque purification and a single amplification on Vero cells (African green monkey kidney; ATCC CCL-81) as previously described [34]. Mosquito bodies and legs were separated and placed in individual tubes with 1 ml mosquito diluent [MD; 20% heat-inactivated FBS in Dulbecco's PBS plus 50 μg/ml penicillin/streptomycin, 50 μg/ml gentamicin, and 2.5 μg/ml Fungizone] plus one 5 mm metal bead (Daisy). Individual samples were thawed and homogenized for 30 seconds at 24 hz in a Mixer Mill MM301 (Retsch), and debris was pelleted by centrifugation at 6000 rcf for 5 minutes and screened or titrated by plaque assay in duplicate on Vero cells as previously described [35].

### Mosquitoes

*Cx. pipiens *egg rafts were originally collected in Pennsylvania in 2004 (courtesy of M. Hutchinson) and subsequently colonized at the Arbovirus laboratory, Wadsworth Center. Mosquitoes were reared and maintained in 30.5 cm^3 ^cages in an environmental chamber at 27°C, 50-65% relative humidity with a photoperiod of 16:8 (light:dark) hours. 400 adult mosquitoes (200 male/200 female) to be used for experiments were collected upon emergence and held in mesh top 3.8 L paper cartons and provided cotton pads with 10% sucrose *ad libitum*. Mosquitoes were held for 4 days to allow for mating.

### Chickens

Day-old, pathogen-free chickens (*Gallus gallus*) were obtained from Charles River (North Franklin, CT) and transferred to the Arbovirus laboratory BSL-3 animal facility in preparation for experimentation. Chickens were housed in metal cages with individual light sources and daily fresh food, water, and resting pads. Three day-old chickens were inoculated subcutaneously with either 10^3 ^pfu WNV in 100 ul animal diluent (endotoxin-free phosphate buffered saline [PBS] +1% fetal bovoine serum [FBS]) or 100 ul diluent alone, 3 days prior to mosquito feeding. All chicken work was approved by the Wadsworth Center Institutional Animal Care and Use Committee (IACUC 06-355).

### Blood feeding

Mosquitoes were deprived of sucrose for 48 hours prior to feeding on chickens. Following starvation, female mosquitoes were removed from the large carton and distributed into two 0.6 L cups for experimental infections in the BSL-3 animal facility. A mock (control) or WNV (experimental) inoculated chicken was placed on top of the mesh of individual cups and carefully restrained manually while mosquitoes were given approximately 1 hour to feed. Following feeding, 100 ul of blood was drawn from the brachial vein of the chickens and transferred to serum separator tubes as previously described [36]. Chicken blood was processed as previously described and serum was saved at -80°C for subsequent plaque titration [9]. Mosquitoes were then anesthetized using CO_2 _and fully-engorged mosquitoes were separated and housed individually in cups containing oviposition dishes with 15 mls of distilled water and with access to 10% sucrose.

Subsequent uninfected blood meals were offered to both control and experimental groups via hanging drops for two days each week of the study. Specifically, mosquitoes were again starved for 48 hrs and then offered 30 ul drops of defibrinated goose blood (Hema Resources) with 2.0% sucrose. Mosquitoes were monitored for one hour during these feedings and both numbers fed and levels of engorgement (1, small amount of blood in abdomen, no abdominal distention; 2, some distention, no pleural membrane observed; 3, significant abdominal blood, pleural membrane observed; 4, fully engorged, distended abdomen) were recorded.

### Mosquito fitness

Survival, wing length, fecundity, and egg hatching were evaluated in this study. Mortality and egg production were monitored and recorded daily for all groups. Wings were removed from dead mosquitoes, individually mounted on slides with double-sided tape, and measured as previously described using a Zeiss microscope, Axiocam camera, and Axiovision software (Carl Zeiss; [9]). Mosquito bodies and legs were processed and tested as described above. Egg rafts were photographed under 50X magnification using a Nikon digital camera (Nikon) and individual eggs were counted using Photo Studio (ArcSoft).

Oviposition cups containing rafts were held for approximately 2 days at 27°C to allow for hatching and 1^st ^instar larvae were counted in order to calculate egg hatching rates.

### Data analysis

Survival curves were generated and analyzed using GraphPad Prism software version 4.0. Comparisons of curves both among groups [(resistant (exposed and uninfected), susceptible (exposed and infected), and unexposed (fed on uninfected chicken)] and between replicates were done with a log-rank test, which is equivalent to the Mantel-Haenszel test. Resistance in this study is defined only as an inability to become infected under these experimental conditions and therefore does not necessarily imply resistance at higher WNV doses. Both survival and reproductive data were used to construct life history tables for each group in separate replicates. Specific calculations included survival (l_x_), equivalent to the proportion of mosquitoes surviving to day x, and reproductive output (m_x_), equivalent to the number of eggs produced on day x. Data for m_x _was smoothed by averaging an individual daily egg output with the egg output on both previous and subsequent days. Subsequent calculations for net reproductive rate (total eggs produced in an average females lifetime; R_0 _= ∑ l_x _m_x_), generation time (average age at which a females lays her eggs; T = ∑ l_x _m_x_x/R_0_), and intrinsic rate of increase (instantaneous population growth rate; r = ln R_0_/T) [37,38] were performed. GraphPad 4.0 was used to construct contingency tables and perform subsequent Fisher's exact and Chi-squared tests for both female egg ovipositing and egg hatch rate among groups. Microsoft Excel was used to perform t-tests for comparisons of mean eggs/raft and levels of engorgement among groups.

## Authors' contributions

All authors have read and approved the final manuscript. ATC analyzed and interpreted the data and wrote the manuscript. LMS designed and carried out the experiments. MAM carried out the experiments. LDK conceived and coordinated the experiments.
